# Supplementary material for: Higher Prevalence of Food Insecurity and Psychological Distress among International University Students during the COVID-19 Pandemic: An Australian Perspective
Source: Int J Environ Res Public Health. 2022 Oct 28;19(21):14101. doi: 10.3390/ijerph192114101 (PMC9658209; doi:10.3390/ijerph192114101)
Supplement: Supplementary file 1 [file ijerph-19-14101-s001.zip › ijerph-1913271-supplementary.pdf]

International Journal of
Environmental Research and Public Health

Supplementary Materials 1

Please specify your gender.

- ○ Male
- ○ Female
- ○ Other
- ○ Prefer not to say

Please specify your age.

____

Are you...?

- ○ An Australian citizen
- ○ A permanent resident
- ○ A visa holder
- ○ Other (please specify) ____

Do you identify as Aboriginal or Torres Strait Islander?

- ○ Yes
- ○ No

What is the main language you speak at home?

- ○ English
- ○ Mandarin
- ○ Cantonese
- ○ Arabic
- ○ Vietnamese
- ○ Italian
- ○ Greek
- ○ Hindi
- ○ Spanish
- ○ Tagalog/Filipino
- ○ Punjabi
- ○ Other (please specify) ____

Are you currently living in Australia?

- ○ Yes
- ○ No

Please answer whether the following statement is often true, sometimes true or never true for you/your household.

In the last 12 months...

	Never true	Sometimes true	Often true	Don't know/prefer not to answer
The food that I bought just didn't last, and I didn't have money to get more	○	○	○	○
I couldn't afford to eat balanced meals	○	○	○	○

In the last 12 months, did you ever cut the size of your meals or skip meals because there wasn't enough money for food?

○ Yes

○ No

○ Don't know/prefer not to answer

Display This Question:

If In the last 12 months, did you ever cut the size of your meals or skip meals because there wasn't... = Yes

How often did this happen?

○ Almost every month

○ Some months but not every month

○ Only 1 or 2 months

○ Don't know/prefer not to answer

In the last 12 months, did you ever eat less than you felt you should because there wasn't enough money for food?

- ○ Yes
- ○ No
- ○ Don't know/Prefer not to answer

In the last 12 months, were you ever hungry but didn't eat because there wasn't enough money for food?

- ○ Yes
- ○ No
- ○ Don't know/Prefer not to answer

We would like to ask you a few questions about your study.

What faculty are you enrolled in?

- ○ Faculty of Science and Engineering
- ○ Faculty of Medicine, Health and Human Sciences
- ○ Faculty of Arts
- ○ Macquarie Business School
- ○ Macquarie University International College

Are you enrolled in...?

- ○ PhD
- ○ Postgraduate study (Masters/Post Graduate Diploma)
- ○ Undergraduate study (Bachelors Degree)
- ○ Post-School Qualification (VET/Diploma/Associate Degree)
- ○ Other (please specify) ____
- ○ I would prefer not to answer this question

Display This Question:

If Are you enrolled in...? = Postgraduate study (Masters/Post Graduate Diploma)

Are you enrolled in...?

- ○ Master of Public Health
- ○ Other (please specify) ____

Display This Question:

If Are you enrolled in...? = Undergraduate study (Bachelors Degree)

Or Are you enrolled in...? = Postgraduate study (Masters/Post Graduate Diploma)

At which stage of the course are you?

- ○ Year 1
- ○ Year 2
- ○ Year 3
- ○ Year 4 or more

Please indicate your student status.

- ○ Domestic
- ○ International
- ○ Exchange

Please indicate your enrolment status.

- ○ Full-time student
- ○ Part-time student

Thinking about your studies in the **last semester...**

	Yes	No	N/A
I managed to plan my studies so that they were done on time	○	○	○
I needed to invest more time and/or effort than usual to do my course-work	○	○	○
I sometimes did nothing, while I should have been doing my coursework	○	○	○
I did really well in my studies	○	○	○
I had the resources I need to do my coursework well (e.g. technology, support from others etc.)	○	○	○
I had a clear understand-ing of what I was expected to do	○	○	○
I had difficulty keeping up	○	○	○

We would like to ask you a few questions about your work.

What **BEST** describes the nature of your current employment situation?

- ○ Employed full-time
- ○ Employed part-time
- ○ Employed casual
- ○ Other (please specify) ____
- ○ Not currently employed
- ○ I would prefer not to answer this question

Display This Question:

If What BEST describes the nature of your current employment situation? != Not currently employed

And What BEST describes the nature of your current employment situation? != I would prefer not to answer this question

Have you been working **in the past two weeks?**

- ○ Yes, I have been working in the past two weeks
- ○ No, I have not been working in the past two weeks (e.g. because I am on leave, business is slow/temporarily closed, etc.)
- ○ I would prefer not to answer this question

We would like to ask you some questions about how COVID-19 has affected your circumstances.

As a result of COVID-19...?

	Yes	No	N/A
I have lost my job	○	○	○
I have started a new job	○	○	○
I have enrolled in a new course of study	○	○	○

Are you currently receiving any COVID-19 related support (e.g. Centrelink or ATO)?

○ Yes

○ No

○ Not yet, but I intend to

Optional: What type of financial support have you received, if any, to help manage the impact of this pandemic?

____
____
____
____
____

Do you have a mental health condition?

○ Yes

○ No

○ Not sure

○ I would prefer not to answer this question

Display This Question:

If Do you have a mental health condition? = Yes

To the best of your knowledge, has a doctor or healthcare professional advised you that you have...?

- ○ An anxiety disorder (e.g. generalised anxiety)
- ○ A depressive disorder (e.g. major depressive disorder)
- ○ Other diagnosis (Please specify) ____
- ○ I would prefer not to answer this question

Display This Question:

If Do you have a mental health condition? = Yes

Have you been able to access the healthcare/medication required for treating your mental health condition during this pandemic?

- ○ Yes, I was able to access treatment as usual
- ○ Yes, I was able to access an alternative (e.g. Telehealth)
- ○ No
- ○ I don't know/ Prefer not to answer

Display This Question:

If Please indicate your student status. = International

If it was necessary, have you been able to access your Overseas Student Health Cover during this time?

- ○ Yes
- ○ No
- ○ Don't know

Display This Question:

If Please indicate your student status. = International

What type of support do you think should be in place to help international students manage the impact of this pandemic?

____

____

____

How would you rate your sleep quality in the **past two weeks?**

- ○ Very poor
- ○ Poor
- ○ Fair
- ○ Good
- ○ Very good

How often did you get enough sleep in the **past two weeks?**

- ○ Never
- ○ Rarely
- ○ Sometimes
- ○ Most of the time
- ○ Always

To what extent did you adhere to a regular sleep routine in the **past two weeks?**

- ○ Not at all
- ○ A little
- ○ A moderate amount
- ○ A lot
- ○ Completely

The following questions relate to how you see yourself and what you have been feeling recently. Please note that your responses to these questions are confidential and you do not need to share any information if you feel uncomfortable in doing so.

The following questions are about your feelings in the **past 30 days**. In the past 30 days, how often did you feel...?

	None of the time	A little of the time	Some of the time	Most of the time	All of the time
Tired out for no good reason	○	○	○	○	○
Nervous	○	○	○	○	○
So nervous that nothing could calm you down	○	○	○	○	○
Hopeless	○	○	○	○	○
Restless or fidgety	○	○	○	○	○
So restless that you could not sit still	○	○	○	○	○
Depressed	○	○	○	○	○
So depressed that nothing could cheer you up	○	○	○	○	○
That everything was an effort	○	○	○	○	○
Worthless	○	○	○	○	○
